# Atrophy in the Left Amygdala Predicted Drug Responses in Idiopathic Generalized Epilepsy Patients With Tonic–Clonic Seizures

**DOI:** 10.3389/fnins.2021.640016

**Published:** 2021-03-31

**Authors:** Xin Li, Zhongyuan Wang, Qian Chen, Xiaoyun Wang, Zhao Qing, Wen Zhang, Jiaming Lu, Junxia Wang, Xin Zhang, Jiani Liu, Zhengge Wang, Baoxin Li, Bing Zhang

**Affiliations:** ^1^Department of Radiology, Nanjing Drum Tower Hospital Clinical College of Nanjing Medical University, Nanjing, China; ^2^Department of Neurology, The Affiliated Drum Tower Hospital of Nanjing University Medical School, Nanjing, China; ^3^Department of Radiology, The Affiliated Drum Tower Hospital of Nanjing University Medical School, Nanjing, China

**Keywords:** drug-resistant epilepsy, generalized tonic–clonic seizures, amygdala, vertex-based shape analysis, support vector machine

## Abstract

We aimed to determine the alterations in the subcortical structures of patients with idiopathic generalized epilepsy with tonic–clonic seizures (IGE-GTCS) *via* MRI volumetry and vertex-based shape analysis and to evaluate the relationships between MRI measures and drug responses. In a follow-up sample of 48 patients with IGE-GTCS and 48 matched normal controls (NCs), high-resolution 3D T_1_WI was performed at baseline. After 1 year of follow-up, 31 patients were classified as seizure free (SF) and 17 as drug resistant (DR). The volumes of subcortical structures were extracted, and vertex-based shape analysis was performed using FSL-Integrated Registration and Segmentation Toolbox (FSL-FIRST). Comparisons among groups were calculated adjusting for covariates [age, sex, and intracranial volume (ICV)]. Analysis of the relationships among imaging biomarkers along with frequency and duration was assessed using partial correlations. The differential imaging indicators were used as features in a linear support vector machine (LSVM). The DR group displayed significant regional atrophy in the volume of the left amygdala compared with NCs (*p* = 0.004, false discovery rate corrected) and SF patients (*p* = 0.029, uncorrected). Meanwhile, vertex-based shape analysis showed focal inward deformation in the basolateral subregion of the left amygdala in DR compared with the results for SF and NC (*p* < 0.05, FWE corrected). There were significant correlations between the volume changes and seizure frequency (*r* = −0.324, *p* = 0.030) and between shape (*r* = −0.438, *p* = 0.003) changes and seizure frequency. Moreover, the volume of the left thalamus in the DR group was significantly correlated with seizure frequency (*r* = −0.689, *p* = 0.006). The SVM results revealed areas under the receiver operating characteristic curve of 0.82, 0.68, and 0.88 for the classification between SF and DR, between SF and NC, and between DR and NC, respectively. This study indicates the presence of focal atrophy in the basolateral region of the left amygdala in patients with IGE drug resistance; this finding may help predict drug responses and suggests a potential therapeutic target.

## Introduction

Idiopathic generalized epilepsy (IGE) with tonic–clonic seizures (GTCS), one of the main genetic generalized epilepsy syndromes, representing approximately 15–20% of all epilepsies (Jallon and Latour, 2005), typically responds well to antiepileptic drug (AED) treatment. However, approximately one-third of patients still develop drug resistance (Brodie et al., 2012) despite the availability of over 20 new AEDs in the past 30 years (Engel and Pitkanen, 2020). Compared with the epilepsy control group, the risk of mortality, dysfunction, and premature death in drug-resistant (DR) epilepsy patients was notably higher, which created a huge burden for patients and the society (Jerome Engel, 2016; Beghi et al., 2019). IGE appears normal on conventional magnetic resonance imaging (MRI) with a diffuse mechanism of seizure onset and no identifiable pathogenesis other than hereditary susceptibility. Identifying common biological disease pathways may help clarify diagnostic and prognostic biomarkers, which in turn helps optimize individual treatment (Pitkänen et al., 2016). Consequently, we aimed to identify imaging biomarkers to predict prognosis in IGE patients.

With the application of non-invasive neuroimaging technology, the understanding of the epilepsy mechanism is improving; furthermore, this technology explores different aspects, such as structural functional or metabolic modification. Structural changes are a prominent feature of many epilepsy types. Based on abundant human and animal neuropathologic, imaging, and electrophysiologic evidence, it has been suspected that a complex cortical–subcortical interaction is the basis of the epileptic process and that subcortical structures (such as the thalamus and basal ganglia) play a crucial role in the generation and propagation of epilepsy (Badawy et al., 2013). Some studies have suggested that electrical stimulation of subcortical structures may exert control on seizure generators initiating epileptic activities, although the mechanism of action remains to be fully elucidated (Zangiabadi et al., 2019). Therefore, the alterations of subcortical nuclei are worth studying. Today, the results of previous studies in subcortical structures in IGE remain controversial. Extensive brain subcortical structural atrophy across several regions, including the thalamus, hippocampus, pallidum, and putamen, was previously reported in IGE patients *via* volumetric and voxel-based morphology (VBM) analysis (Betting et al., 2006; Bernhardt et al., 2009; Du et al., 2011; Whelan et al., 2018). Some studies have found no significant difference in the subcortical structure volume of IGE-GTCS patients compared with that of normal controls (NCs) (Bernasconi et al., 2003; Natsume et al., 2003; Seeck et al., 2004; Ozturk et al., 2020). Some functional MRI studies complemented these findings, indicating connectome anomalies in the thalamus and hippocampus (Wang et al., 2019a,b). Despite the contribution of these findings to localizing anomalies in this disease, there is limited insight into the specific clinical value of the abnormality of subcortical nuclei. Hence, we attempt to explore more subtle forms of structural damage in the subcortical structures from patients with different drug responses and to predict drug efficacy in IGE-GTCS patients.

Complementary to VBM, vertex-based shape analysis is an automatic method that provides useful information about the location and pattern of morphological changes in subcortical structures. Shape analysis is now widely used to evaluate the regional atrophy of subcortical structures in various neurological and psychiatric disorders (Zarei et al., 2010; Gelineau-Morel et al., 2012). Moreover, combining volumetric and morphological analyses will improve the accuracy and sensitivity of structural alterations.

In the present study, we hypothesized that IGE-GTCS patients with different drug responses would have diverse changes in the subcortical nuclei, and the structural alteration of subcortical nuclei may be potential biomarkers of drug responses in IGE patients. To verify this hypothesis, we conducted both volume and morphology analyses of subcortical structures with FSL-Integrated Registration and Segmentation Toolbox (FSL-FIRST) software in IGE-GTCS patients to determine specific patterns that might be a biomarker to predict which patients will develop drug resistance.

## Materials and Methods

### Subjects

**FIGURE 1:**
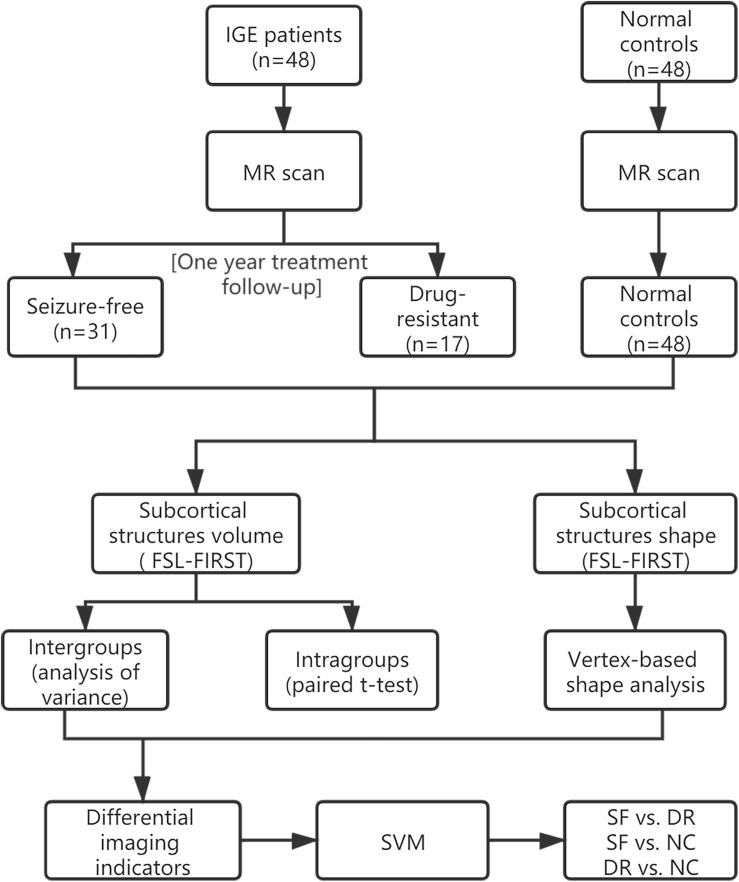
Schematic figure of the analytical methods.

In order to make it easier for readers to understand, we made a schematic figure of the analytical methods (Figure 1). Forty-eight consecutive right-handed patients with IGE-GTCS admitted to the Nanjing Drum Tower Hospital from 2013 to 2019 were enrolled in the study. They met the following inclusion and exclusion criteria: (1) those diagnosed with idiopathic/hereditary generalized epilepsy with only GTCS according to the current International League Against Epilepsy (ILAE) classification of seizure types (Fisher et al., 2017) based on electroclinical symptomatology were included (we ruled out patients with myoclonic or absence seizures to exclude patients with juvenile myoclonic epilepsy or juvenile absence seizures); (2) subjects who had psychiatric disorders, brain structural lesions, systemic disease, or other MRI contraindications were excluded; (3) prospective clinical treatment follow-up of at least 1 year after an imaging investigation was required for inclusion; and (4) participation in a 3-T study MRI on the same scanner and 3D T_1_WI results were necessary for inclusion.

In patients, we established the drug response *via* the ILAE criteria (Kwan et al., 2010) at least at a 1-year follow-up point after the imaging scan and divided patients into seizure-free (SF) and DR groups. After 1 year of follow-up, 31 patients (11 females, mean ± SD age = 24.11 ± 10.62 years) were classified as SF, and 17 patients (9 females, mean ± SD age = 28.53 ± 9.91 years) were classified as DR. Of these patients, 15 SF patients and 3 DR patients had not taken medication before imaging (mean ± SD duration = 1.73 ± 2.08 years), and 30 patients (16 SF and 14 DR) were treated with monotherapy or polytherapy including valproate (VPA), levetiracetam (LEV), lamotrigine (LTG), oxcarbazepine (OXC), topiramate (TPM), carbamazepine (CBZ), phenytoin (PHT), clonazepam (CZP), and phenobarbital (PB) before the study (mean ± SD duration = 9.45 ± 7.29 years). The information of the drug exposure prior to MRI scan and treatment selection after enrollment is in Supplementary Table 2 (SocioDemographic).

Forty-eight age-matched and sex-matched NCs (17 females, mean ± SD age 25.63 ± 3.25 years) were enrolled during the same recruitment period. They underwent the same image scanning with the same quality control standards as the patients. Prior to the study, all subjects (patients and NCs) gave written informed consent, and this study was approved by the Research Ethics Committee of Nanjing Drum Tower Hospital.

### MRI Acquisition

Brain scanning of all the subjects was performed on a Philips Healthcare (Best, the Netherlands) 3 T MR imaging scanner with an eight-channel head coil. High-resolution 3D T_1_WI was conducted using a 3D turbo field echo sequence (repetition time 9.8 ms, echo time 4.6 ms, inversion time 900 ms, flip angle 8°, voxel size 1.0 mm × 1.0 mm × 1.0 mm, and 192 slices). During the examination, the subjects were ordered to close their eyes and remain motionless.

### Image Analysis

To investigate the alterations in subcortical structures, we first applied the DARTEL algorithm in the SPM12 toolbox^1^ to preprocess each anatomical image to perform intensity correction and skull dissection. A Bayesian model-based segmentation tool in FSL-FIRST^2^ was used to segment subcortical nuclei (bilateral thalamus, hippocampus, amygdala, nucleus accumbens, putamen, caudate, and palladium), cerebrospinal fluid (CSF), gray matter (GM), and white matter (WM). No participant was excluded owing to poor structural segmentation, following FSL-FIRST guidelines^3^. Then, we calculated the volumes of the individual subcortical nuclei and the intracranial volume (ICV). For the subcortical nuclei of all subjects, the vertex index was calculated using the FSL vertex analysis script, *first_utils script*, which is based on the signed vertical distance from the corresponding surface mesh (a vtk file produced by using the FSL *run_first_all script*) in the Montreal Neurological Institute (MNI) template. A positive index indicated outward deformation or expansion of the surface of a given structure, whereas a negative index indicated inward deformation or atrophy of the surface of the structure. Finally, the degree of deformation value for all participants was computed and then used for statistical analysis.

### Statistical Analysis

This study used IBM SPSS Statistics, Version 23.0, to determine a statistical description and make statistical inferences. A chi-square test was used to assess the classification variables. Analysis of variance (ANOVA) was performed to evaluate continuous variables. ANOVA was used for between-group comparisons (SF vs. DR, SF vs. NC, DR vs. NC, SF vs. DR vs. NC) of the volume of subcortical nuclei after adjusting the covariates (age, sex, and ICV). An analysis with *a priori* determination of the significance level at *p* < 0.05 was considered statistically significant, false discovery rate corrected (FDR corrected). The volume difference between the left and right amygdala was compared by using paired *t*-test in the three groups (SF, DR, and NC) separately. Intergroup differences in subcortical shape were assessed using a method based on a non-parametric approach for seven pairs of nuclei using the FSL randomization procedure and adjusting the covariates (age and sex). The findings were corrected for multiple comparisons using threshold-free cluster enhancement (TFCE) with a familywise error (FWE) rate of *p* < 0.05 by running 5,000 random permutations. Then, after controlling for age, sex, and ICV, the relationship between the volume and shape of the structures with seizure frequency and disease duration was analyzed by partial correlation.

### SVM-Based Classification

To explore whether imaging indicators can be used to classify including SF vs. DR, DR vs. NC, SF vs. NC, we applied the LIBSVM toolbox for MATLAB to implement the linear support vector machine (LSVM) classification^4^. LSVM is one of the most widely used supervised machine learning methods (Cui et al., 2016). Its purpose is to obtain a classifier with high predictive ability by minimizing the empirical classification error on the training data while considering the complexity of the model. In this study, the SVM toolbox in MATLAB was used to verify the role of the morphology and volume of subcortical structures in the classification of subjects. Leave-one-out cross-validation (LOOCV) was adopted; that is, each subject was separately used as the test set in turn, and the remaining subjects belonging to the contrast group were used as the classifier to form the training set. We used the SVM with the linear kernel function, and the parameter *C* was set to 1. Discriminative features were derived from differential imaging indicators of seven pairs of subcortical structure volumes and shapes. The accuracy, sensitivity, specificity, and operating curve (ROC) of the classifier in the classification of test set data in each test were recorded. Then, the area under the curve (AUC) of the ROC was statistically compared based on the DeLong method. To test the sample size imbalance effect on the classification, a control analysis to select the same sample size of DR (*n* = 17) from NC (*n* = 48) was performed.

## Results

### Clinical Features

Anatomical MRI data from 96 participants were included in the study, consisting of 31 SF patients, 17 DR patients, and 48 NCs. The demographic and clinical characteristics of the subjects are listed in Table 1. The baseline features of sex and age among the three groups (all *p* > 0.05) were generally well balanced. Patients who were not SF indeed had a higher seizure frequency (*t* = 19.700, *p* = 0.000) and had a longer illness duration (*t* = 23.896, *p* = 0.000) than SF patients. However, there was no significant difference in onset age between the two groups (*t* = 0.035, *p* = 0.852).

**TABLE 1:** Demographic and clinical characteristics of the subjects.

**Characteristics**	**SR (*n* = 31)**	**DR (*n* = 17)**	**NC (*n* = 48)**	***F* or *t***	***p***
**Demographic**					
Age (years)	24.11 ± 10.62	28.53 ± 9.91	25.63 ± 3.25	1.788	0.173
Sex (F/M)	11/20	9/8	17/31	1.808	0.405
**Medical history**					
Duration (years)	4.81 ± 4.54	9.74 ± 9.35	/	19.700	0.000*
Frequency^#^ (times/years)	1.90 ± 1.55	7.06 ± 7.02	/	23.896	0.000*
Onset age (year)	19.35 ± 10.33	18.80 ± 8.78	/	0.035	0.852

SF, seizure free; DR, drug resistant; NC, normal control.

*There were statistically significant differences between the groups (p < 0.05).

*^#^Numbers of generalized tonic–clonic seizure per year.*

### MRI Features

#### MR Volumetry

**FIGURE 2:**
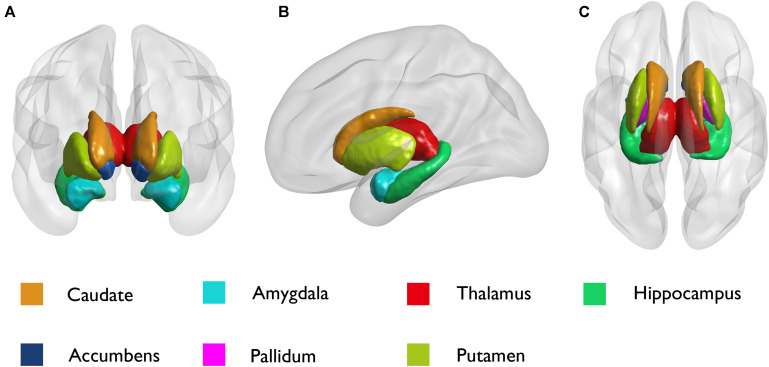
Schematic diagram of subcortical structures by the BrainNet Viewer. **(A)** Coronal; **(B)** sagittal; and **(C)** axial.

**FIGURE 3:**
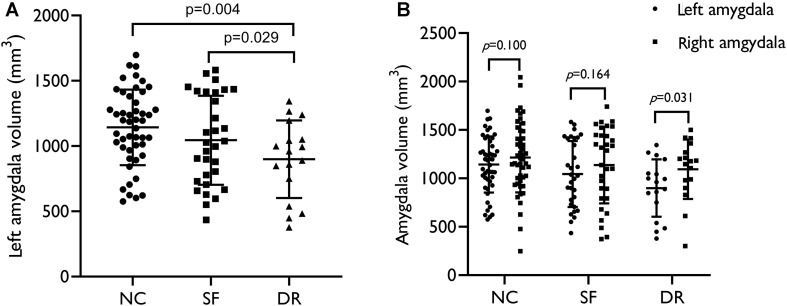
**(A)** Volume distribution of the left amygdala in patients and healthy controls. **(B)** Intragroup volume of the bilateral amygdala. SF, seizure free; DR, drug resistant; NC, normal control. The results were corrected for sex, age, and ICV (*p* < 0.05).

We analyzed seven pairs of subcortical nuclei, including the bilateral thalamus, hippocampus, amygdala, nucleus accumbens, putamen, caudate, and palladium. The subcortical nuclei schematic is shown in Figure 2. There were significant differences in the volume of the left amygdala between the three groups (*p* = 0.008, uncorrected) (Table 2). Compared with NCs, DR patients showed a distinct decrease in the volume of the left amygdala (*p* = 0.004, FDR corrected, Figure 3A), and compared with SF patients, the volume of the left amygdala was reduced in DR patients (*p* = 0.029, uncorrected). There was no difference in the volume between SF patients and NCs (*p* > 0.05). Additionally, there was no significant difference in bilateral amygdala volume in NCs (*p* = 0.100) and SF patients (*p* = 0.164), but the volume of the left amygdala was smaller than that of the right amygdala in the DR group (*p* = 0.031) (Figure 3B and Supplementary Table 1). Interestingly, the thalamus and hippocampus did not show any significant alterations. Moreover, there was no significant difference in the shape (*p* > 0.05) and volume (*p* = 0.372) of the left amygdala between the medication group and the non-medication group.

**TABLE 2:** Comparison of subcortical structure volumes among groups (*p* < 0.05).

	**SF**	**DR**	**NC**	***F* 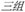 **	***p* 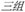 **	**Partial η ^2^ 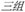 **	***p*_*SF:DR*_**	**Partial η ^2^_*S*__*F:DR*_**	***p*_*SF:NC*_**	**Partial η ^2^_*S*__*F:NC*_**	***p*_*DR:NC*_**	**Partial η ^2^_*D*__*R:NC*_**
Left thalamus	7,412.30 ± 1,245.33	7,582.82 ± 831.17	7,732.31 ± 717.61	0.636	0.532	0.014	0.415	0.016	0.765	0.001	0.380	0.013
Left caudate	3,710.20 ± 492.66	3,438.06 ± 379.91	3,650.69 ± 502.83	1.610	0.206	0.035	0.046	0.089	0.182	0.024	0.536	0.006
Left putamen	5,323.65 ± 933.69	5,166.35 ± 730.23	5,615.11 ± 676.97	0.871	0.422	0.019	0.636	0.005	0.403	0.009	0.260	0.021
Left pallidum	1,654.81 ± 295.36	1,607.29 ± 160.15	1,729.82 ± 194.88	0.642	0.529	0.014	0.635	0.005	0.725	0.002	0.095	0.046
Left hippocampus	3,929.52 ± 700.68	4,039.23 ± 642.66	4,075.09 ± 580.59	0.665	0.517	0.015	0.352	0.020	0.704	0.002	0.241	0.023
Left amygdala	1,044.77 ± 341.27	899.29 ± 296.80	1,143.07 ± 289.25	5.149	0.008*	0.103	0.029*	0.106	0.382	0.010	0.004*	0.132
Left accumbens	550.84 ± 161.44	542.41 ± 44.75	592.29 ± 84.47	1.063	0.350	0.023	0.381	0.018	0.749	0.001	0.052	0.061
Right thalamus	7,032.85 ± 1,176.50	7,129.11 ± 832.72	7,336.86 ± 833.64	0.396	0.674	0.009	0.332	0.022	0.697	0.002	0.815	0.001
Right caudate	3,776.58 ± 609.64	3,595.47 ± 390.79	3,746.30 ± 532.47	0.977	0.380	0.021	0.221	0.035	0.204	0.022	0.932	0.000
Right putamen	5,432.91 ± 699.52	5,252.88 ± 772.99	5,632.90 ± 619.48	0.598	0.552	0.013	0.582	0.007	0.538	0.005	0.441	0.010
Right pallidum	1,715.36 ± 280.72	1,654.00 ± 300.58	1,784.27 ± 227.46	0.347	0.708	0.008	0.641	0.005	0.831	0.001	0.482	0.008
Right hippocampus	4,194.58 ± 604.84	4,111.82 ± 733.85	4,375.10 ± 460.88	0.362	0.697	0.008	0.897	0.000	0.504	0.006	0.406	0.012
Right amygdala	1,137.90 ± 395.57	1,094.00 ± 307.51	1,215.08 ± 360.48	0.609	0.546	0.013	0.603	0.006	0.683	0.002	0.283	0.019
Right accumbens	452.16 ± 107.69	431.29 ± 63.03	446.77 ± 87.22	0.939	0.395	0.020	0.334	0.022	0.137	0.030	0.842	0.001

*There were statistically significant differences between the groups (*p* < 0.05).

#### Vertex-Based Shape Analysis

**FIGURE 4:**
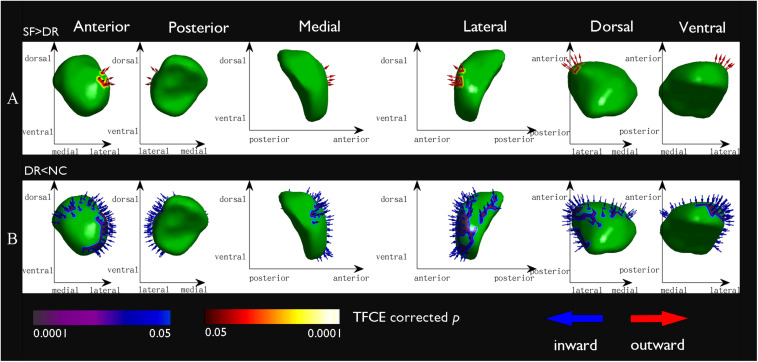
Vertex-based shape analysis results of the comparison between groups at a significance level of *p* < 0.05 (FWE corrected) and controlling for age and sex. Red arrows point outward (expansion) and blue arrows point inward (atrophy). The *x*- and *y*-axes indicate different directions (dorsal, ventral, medial, lateral, anterior, posterior) to show different location information. **(A)** The seizure free > the drug resistant, the basolateral region of the left amygdala: 172/872 19.72%; **(B)** the drug resistant < the normal controls, the basolateral region of the left amygdala: 616/911 67.62%.

Compared with NCs, DR patients showed significant regional atrophy in the shape of the left amygdala (L_Amyg: 616/911 67.62%, *p* < 0.05, FWE corrected), which was located in the basolateral region (Figure 4B). Meanwhile, a similar part of the left amygdala in the DR group was also slightly reduced compared with the amygdala of SF patients (L_Amyg: 172/872 19.72%, *p* < 0.05, FWE corrected) (Figure 4A). In addition, the shape of the left putamen (L_Puta: 94/2,969 3.17%, *p* < 0.05, FWE corrected) showed slight inward deformation (atrophy) in the DR group compared with that of NCs. Furthermore, compared with controls, SF patients had minor inward deformation in the shape of the left pallidum (L_Pall: 15/1,128 1.33%, *p* < 0.05, FWE corrected).

#### Correlation Between MRI Measurements and Clinical Parameters

The correlations between the morphology and volume changes of subcortical nuclei and the seizure frequency and disease duration of IGE are provided in Table 3. The data of all patients showed that seizure frequency was negatively correlated with left amygdala shape (*r* = −0.438, *p* = 0.003, uncorrected) and volume (*r* = −0.324, *p* = 0.03, uncorrected) (Table 3 and Supplementary Table 2). In the DR group, although there was no statistically distinct difference, there was a trend of correlation between the shape and volume of the left amygdala and seizure frequency. Moreover, the volume of the left thalamus in the DR group was significantly negatively related to seizure frequency (*r* = −0.689, *p* = 0.006, uncorrected), but a correlation was not found in all patients. No correlation was found for the disease duration in either patient group. On the other hand, left amygdala volume was positively correlated with right amygdala volume in the NC group (*r* = 0.599, *p* = 0.000) and SF (*r* = −0.522, *p* = 0.003), but this correlation was not found in the DR group (*r* = 0.373, *p* = 0.140) (Supplementary Table 1).

**TABLE 3:** Correlation among volume and shape changes of subcortical nuclei along with seizure frequency and IGE duration.

**Groups**		**DR**				**SF + DR (IGE)**			
**Clinical feature**		**Frequency**		**Duration**		**Frequency**		**Duration**	
**Statistics**		***r***	***p***	***r***	***p***	***r***	***p***	***r***	***p***
Shape	Left amygdala	–0.357	0.211	–0.261	0.367	–0.438	0.003*	–0.265	0.078
Volume	Left amygdala	–0.442	0.114	–0.237	0.415	–0.324	0.030*	–0.226	0.136
	Left thalamus	–0.689	0.006*	–0.527	0.053	–0.154	0.311	–0.182	0.232

*There were statistically significant differences between the groups (the significance level at p < 0.05).

### SVM Classification Results

**FIGURE 5:**
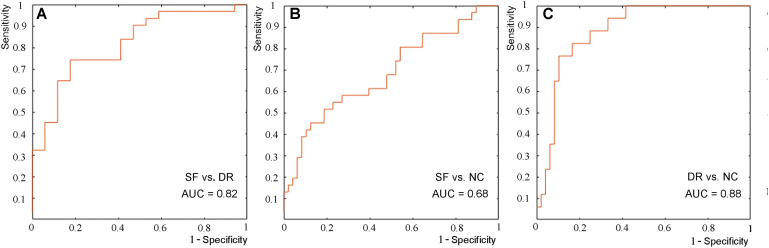
ROC curve of the classification between seizure-free and drug-resistant patients **(A)**, between seizure-free patients and NCs **(B)**, and between drug-resistant patients and NCs **(C)**.

The results of the LSVM classification are shown in Figure 5 and Supplementary Table 3. Discriminative features were derived from age and differential indicators including the volume and shape of the left amygdala and palladium and putamen. The best results are retained for each group classification. The demographical feature of age among the three groups (all *p* > 0.05) was generally well balanced, but the age of our subjects (IGE patients) ranged from 12 to 57 years old. Therefore, age was regarded as a feature in this study. The characteristics that identify SF patients from NCs are age, the shape of the left amygdala and palladium, and volume of the left amygdala; those that differentiate DR patients from NCs are age, the shape of the left amygdala and putamen, and volume of the left amygdala; and those that discriminate SF patients and DR patients include age, shape, and volume of the left amygdala and palladium. LSVM distinguished DR patients from NCs with an accuracy of 84.62%, sensitivity of 58.82%, specificity of 87.50%, and AUC of 0.88, whereas the accuracy, sensitivity, specificity, and AUC for differentiating SF patients from NCs were 70.89, 41.94, 89.58%, and 0.68, respectively. In terms of distinguishing DR patients from SF patients, the accuracy, sensitivity, specificity, and AUC were 77.08, 90.32, 52.94%, and 0.82, respectively. Furthermore, LSVM distinguished DR patients (*n* = 17) from NCs (*n* = 17) with an accuracy of 82.35%, sensitivity of 82.35%, specificity of 82.35%, and AUC of 0.90, and the classification features are the same as above (Supplementary Figure 1).

## Discussion

In this study, alterations in subcortical nuclei in IGE-GTCS patients with different drug responses were investigated by MR volume and vertex-based shape analysis. Both volume and morphology analysis of subcortical structures showed atrophy in the basolateral region of the left amygdala in DR patients. Moreover, there were significant correlations between the volume and shape changes and between the volume and seizure frequency. The correlation of bilateral amygdala volume was not present in the DR group. The volume of the left thalamus in the DR group was significantly related to seizure frequency. The LSVM results revealed AUCs of 0.8159, 0.6848, and 0.8811 for the classification between SF and DR patients, between SF patients and NCs, and between DR patients and NCs, respectively.

### Atrophy of the Left Amygdala, Especially in Basolateral Areas Contributed to Drug Resistance

It is well known that the amygdala is regarded as part of the limbic system of the brain and is subdivided into three groups comprised of the basolateral complex, the corticomedial nucleus, and the central nucleus. The amygdala plays a crucial role in many physiological and pathological mechanisms, including emotion regulation, memory organization, epileptogenicity, and others (Janak and Tye, 2015). In this study, pharmacoresistant patients had more frequent seizures than SF patients. There is growing evidence that seizure-induced alterations could also be generated by recurrent seizures, especially in structures vulnerable to damage (e.g., the amygdala or hippocampus) (Duncan, 2002; Lopim et al., 2016). One study has shown that stimulating the right amygdala can cause negative emotions, especially fear and sadness, and stimulating the left amygdala can cause pleasant or unpleasant (fear, anxiety, and sadness) emotions (Lanteaume et al., 2007). It is reasonable to speculate that repeated seizures induce bad experiences and increase negative emotions in DR patients, resulting in damage to the left amygdala. Accumulating evidence suggests that an accelerated loss of neurons in the epileptogenic zone has conventionally been considered to be a factor associated with poor prognosis in epilepsy (Salmenpera et al., 2001). Furthermore, previous studies have shown that neurons in the basolateral amygdala consist of lateral (LA), basal (BA), and basomedial (BM) cell groups and play an important role in associative learning (Zhang and Li, 2018). The basolateral subregion receives information on the external environment from the sensory thalamus and sensory cortex, which strongly project to the LA. The basolateral amygdala is interconnected with sensory contact areas and cortical areas, especially the hippocampus, midline, and orbital prefrontal cortex (McDonald, 1998; Solano-Castiella et al., 2010). In our research, the contraction in the basolateral area as a functional area of the amygdala was more compelling than other findings. Meanwhile, substantive evidence has shown that thalamocortical networks play an essential role in GTCS (Bernhardt et al., 2009). Notably, there are complex fiber projections between the thalamus, amygdala, and hippocampus (McDonald and Mott, 2017; Aizenberg et al., 2019). Consequently, shrinkage of the left amygdala in DR patients can be understood and explained. A study showed that chronic epilepsy and memory impairment in patients with DR medial temporal lobe epilepsy (TLE) are related to different patterns of histopathological changes in the hippocampus, amygdala complex, and entorhinal region. Major histopathological alterations included neuronal cell loss and cellular and fibrillary gliosis in the lateral and basal nuclei of the amygdala. Patients with secondary GTCS showed more serious damage in these areas (Yilmazer-Hanke et al., 2000). It is universally accepted that the amygdala kindling model in fully kindled rats may be an available model for DR patients with complex partial seizures with secondary generalization (Loscher et al., 1986; Welzel et al., 2019). On the other hand, some studies have demonstrated that the amygdala and amygdala pathways can predict the treatment outcome of social anxiety disorder and affect therapeutic effects (Klumpp and Fitzgerald, 2018). Experimental evidence suggests that emotional stimuli can influence many different aspects of cognition and behavior, resulting in disrupted cognitive goals and less optimal task performance (Vuilleumier et al., 2001; Ladouceur et al., 2018). Emotional processing and regulation interact with treatment in a number of diseases. It is well established that epilepsy is a common neurological disorder that can be complicated with neurobehavioral comorbidities, including cognitive disorders and psychiatric disorders (Hermann et al., 2008; Lin et al., 2012). As a result, our finding that patients with poor drug responses have atrophy in the left amygdala, especially in basolateral areas, is reasonable. Brain subcortical structural changes across several regions, including the thalamus, hippocampus, pallidum, and putamen, were formerly reported in IGE patients (Badawy et al., 2013; Kim et al., 2013; Pitkänen et al., 2016). Previous studies have not found obvious amygdala changes, which may be due to the lack of subgroup analysis of drug responses. On the other hand, we did not find a difference between the thalamus and hippocampus, which may be due to insufficient obvious differences, the influence of drug treatment, the degree of social support, etc. Furthermore, the vertex-based morphological analysis showed slight atrophy of the left pallidum and putamen in patients, which is consistent with the findings in previous studies (Pitkänen et al., 2016; Beghi et al., 2019). This shows that the left pallidum and putamen may play an unknown role in IGE-GTCS.

Notably, the volume and shape atrophy in the left amygdala found in the between-group comparison was negatively correlated with seizure frequency, suggesting that basolateral regions of the amygdala are preferentially affected in IGE patients. As a clinical index of epilepsy, seizure frequency can indirectly reflect the severity of the disease. The negative correlation indicated that the more frequent seizures were, the more obvious the damage to the amygdala in IGE-GTCS patients was. In the DR group, although there was no obvious relevance in clinical indicators and imaging indicators, there was a trend in relativity. This may be because the sample size was too small to achieve a statistically significant difference. The correlation of bilateral amygdala volume was not present in the DR group. This may indicate that changes in the amygdala are related to drug resistance. In addition, the volume of the left thalamus in the DR group was significantly negatively related to seizure frequency. This correlation was not found in the SF group or among all patients. This may suggest that the left thalamus is related to drug resistance. Several studies have suggested that the anterior thalamic region (ANT) is vital to the maintenance and propagation of seizures explained by its extensive connections (Bouwens van der Vlis et al., 2019). The ANT is the most widely used target for deep brain stimulation (DBS) in the treatment of DR epilepsy (Fisher et al., 2010). These findings may reveal the relationship between disease severity and imaging indicators, which will help improve the present understanding of the emergence of drug resistance, predict DR patients, and provide an advanced therapeutic target for DR epilepsy.

### Classification Performance of the SVM

We explored the classification results of drug responses in IGE-GTCS patients using LSVM with imaging indicators. Although the results are not very satisfactory, they provided us with an opportunity to judge the efficacy of the drug clinically. This is a brave attempt in the study of prognostic biomarkers in DR epilepsy. In the future, this needs to be verified in animal experiments and brain connectomics studies. The mediocre performance may have been due to the following reasons. First, our sample size was not large enough though it was essential for classification performance, and the sample size imbalance effect was also a source of poor classification results. In addition, amygdala atrophy may have a complex pathophysiological mechanism and may not be suitable for classification. Our findings support the clinical validity of left amygdala contraction as a potential means for clinicians to predict the efficacy of a drug in IGE patients who have no obvious lesions on conventional MRI.

### Limitations and Settlements

The main limitation of our study is that the sample size of the patients in the DR group was small. This may cause the statistical effect to be erroneously low. For example, the difference in the left amygdala volume between the DR group and the SF group was not corrected for multiple comparisons. Clinical and imaging indicators had related trends, but no significant difference was found. Moreover, a larger sample size can provide more representative features for obtaining a more stable and reliable classification performance. However, the recruitment of DR subjects is still challenging owing to the low incidence and the demands of follow-up work. Second, although the mechanism of the effect of AEDs on brain structure is unclear, we cannot eliminate this drug effect. Some subjects had already taken AEDs before the study began. Some studies have shown that AEDs can affect a person’s brain structure, resulting in pseudoatrophy of the brain or neurogenesis (Papazian et al., 1995). On the positive side, we examined the changes in the nuclei in patients who were taking medication at baseline and those who were not. There was no significant difference in the shape and volume of the left amygdala between these two groups. We can cautiously say that the drug may not have a significant effect on the left amygdala in our study. Moreover, we did not perform a psychiatric evaluation, so we cannot determine the patients’ mental state to further verify our results. Finally, although we obtained relatively good classification accuracy by LSVM based on the volume and shape analysis of subcortical structures, it is still not suitable to serve as a substitute for traditional clinical methods for evaluating drug resistance. In the future, more subjects need to be included in studies using more advanced classifiers to further validate our findings. Hopefully, more effective and high-quality imaging indicators can be provided for the clinic, which can identify DR patients, thereby reducing drug side effects and improving the quality of life of patients by increasing social support and care.

## Conclusion

The findings from this study suggested the presence of focal atrophy of the left amygdala located in the basolateral region, which may help predict drug response and suggest a potential therapeutic target such as DBS.

## Data Availability Statement

The original contributions presented in the study are included in the article/Supplementary Material, further inquiries can be directed to the corresponding author/s.

## Ethics Statement

The studies involving human participants were reviewed and approved by the Research Ethics Committee of Nanjing Drum Tower Hospital. Written informed consent to participate in this study was provided by the participants’ legal guardian/next of kin.

## Author Contributions

XL, ZYW, and QC contributed to the design and manuscript preparation. JW and JLi were responsible for collecting data. XW, ZQ, and WZ were involved in analyzing the imaging data. JLu and XZ executed the experimental work. ZGW, BL, and BZ guided the design of the study protocol and reviewed and critiqued the manuscript. All authors have read and approved the manuscript.

## Conflict of Interest

The authors declare that the research was conducted in the absence of any commercial or financial relationships that could be construed as a potential conflict of interest.

## Footnotes

1 http://www.fil.ion.ucl.ac.uk/spm/software/spm12

2 http://www.fmrib.ox.ac.uk/fsl/FIRST

3 https://fsl.fmrib.ox.ac.uk/fsl/fslwiki/FIRST/UserGuide

4 http://www.csie.ntu.edu.tw/~cjlin/libsvm/
