# Supplementary material for: Atrophy in the Left Amygdala Predicted Drug Responses in Idiopathic Generalized Epilepsy Patients With Tonic–Clonic Seizures
Source: Front Neurosci. 2021 Mar 31;15:640016. doi: 10.3389/fnins.2021.640016 (PMC8044327; doi:10.3389/fnins.2021.640016)
Supplement: Supplementary file 1 [file Table_1.DOCX]

Table 1｜The difference in and correlation of bilateral amygdala volume among subjects

| Group | Left amygdala volume | Right amygdala volume | *t* | *p* | r | *p* |
| --- | --- | --- | --- | --- | --- | --- |
| SF | 1044.77±341.27 | 1137.90±395.57 | -1.426 | 0.164 | 0.522 | 0.003* |
| DR | 899.29±296.80 | 1094.00±307.51 | -2.372 | 0.031* | 0.373 | 0.140 |
| NC | 1143.07±289.25 | 1215.08±360.48 | -1.676 | 0.100 | 0.599 | 0.000* |

Table 2｜Correlation among volume and shape changes of all subcortical nuclei along with seizure frequency and IGE duration.

| Groups | | DR | | | | SF+DR (IGE) | | | |
| --- | --- | --- | --- | --- | --- | --- | --- | --- | --- |
| Clinical feature | | Frequency | | Duration | | Frequency | | Duration | |
| Statistics | | r | *p* | r | *p* | r | *p* | r | *p* |
| Shape | Left amygdala  Left pallidum | -0.357  0.056 | 0.211  0.850 | -0.261  -0.307 | 0.367  0.285 | -0.438  0.011 | 0.003*  0.940 | -0.265  0.125 | 0.078  0.414 |
| Volume | Left amygdala | -0.442 | 0.114 | -0.237 | 0.415 | -0.324 | 0.030* | -0.226 | 0.136 |
|  | Left thalamus | -0.689 | 0.006* | -0.527 | 0.053 | -0.154 | 0.311 | -0.182 | 0.232 |
|  | Left caudate | -0.350 | 0.220 | -0.029 | 0.922 | -0.189 | 0.214 | 0.125 | 0.413 |
|  | Left putamen | -0.359 | 0.208 | -0,019 | 0.950 | -0.026 | 0.865 | 0.172 | 0.259 |
|  | Left pallidum | -0.068 | 0.817 | -0.061 | 0.836 | 0.028 | 0.855 | 0.215 | 0.155 |
|  | Left hippocampus | -0.219 | 0.451 | 0.034 | 0.909 | -0.006 | 0.970 | -0.109 | 0.478 |
|  | Left accumbens | -0.390 | 0.168 | 0.014 | 0.962 | -0.027 | 0.858 | 0.099 | 0.518 |
|  | Right thalamus | -0.489 | 0.076 | -0.475 | 0.086 | -0.132 | 0.386 | -0.210 | 0.167 |
|  | Right caudate | -0.464 | 0.095 | -0.188 | 0.519 | -0.112 | 0.462 | 0.053 | 0.730 |
|  | Right putamen | -0.465 | 0.094 | -0.109 | 0.712 | -0.128 | 0.402 | 0.066 | 0.665 |
|  | Right pallidum | -0.606 | 0.022* | -0.251 | 0.387 | -0.235 | 0.121 | -0.133 | 0.383 |
|  | Right hippocampus | -0.246 | 0.396 | 0.158 | 0.589 | -0.026 | 0.866 | 0.154 | 0.312 |
|  | Right amygdala | -0.139 | 0.636 | -0.208 | 0.476 | -0.034 | 0.824 | -0.117 | 0.444 |
|  | Right accumbens | -0.156 | 0.594 | 0.022 | 0.940 | 0.004 | 0.978 | 0.006 | 0.968 |

Table 3｜The results of LSVM classification among groups.

| Groups | Accuracy | Sensitivity | Specificity | AUC |
| --- | --- | --- | --- | --- |
| SF vs DR | 77.08% | 90.32% | 52.94% | 0.8159 |
| SF vs NC | 70.89% | 41.94% | 89.58% | 0.6848 |
| DR vs NC | 84.62% | 58.82% | 87.50% | 0.8811 |


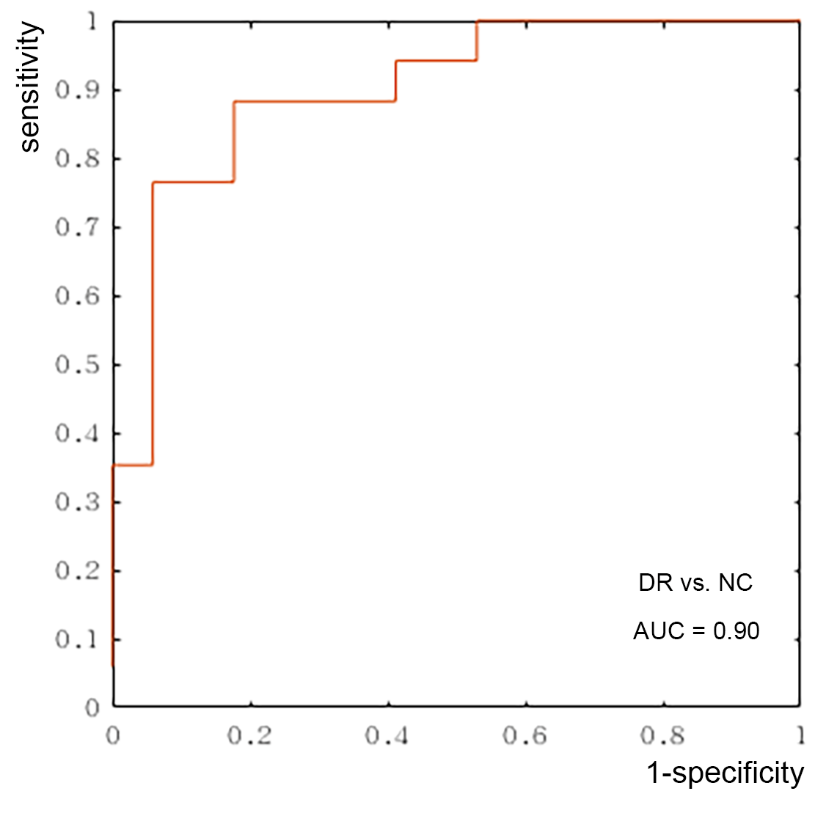


Figure 1｜ROC curve of the classification between drug-resistant patients and NCs (n=17).
